# Virtual screening and molecular dynamics simulation study of ATP-competitive inhibitors targeting mTOR protein

**DOI:** 10.1371/journal.pone.0319608

**Published:** 2025-05-05

**Authors:** Mei-Yu Jin, Hao Yu, Qiong Deng, Zhu Wang, Jie-Yan Wang, Hao-Long Li, Hui Liang

**Affiliations:** 1 Shenzhen Institute of Advanced Technology, Chinese Academy of Sciences, Shenzhen, China; 2 Department of Urology, People’s Hospital of Longhua, Shenzhen, China; 3 State Key Laboratory for Diagnosis and Treatment of Severe Zoonotic Infectious Diseases, Key Laboratory for Zoonosis Research of the Ministry of Education, Institute of Zoonosis, and College of Veterinary Medicine, Jilin University, Changchun, China; King Abdulaziz University, SAUDI ARABIA

## Abstract

In order to explore efficient ATP-competitive mTOR inhibitors and aid the development of targeted anticancer drugs, this study focuses on virtual screening and molecular dynamics simulations. The compounds were sourced from the ChemDiv commercial compound library, and through virtual screening, 50 ligands with favorable binding modes and excellent docking scores were selected from 902,998 compounds. Molecular dynamics simulations, including RMSD (Root Mean Square Deviation) and RMSF (Root Mean Square Fluctuation), were used to further evaluate these 50 ligands. Structural stability, key residue interactions, hydrogen bonding, binding free energy, and other factors were quantitatively and qualitatively analyzed. Top1, top2, and top6, which exhibited outstanding performance, were identified. Simulations revealed that they bind stably in the active region of the mTOR protein, forming hydrogen bonds, π-π interactions, and hydrophobic interactions with key amino acid residues such as VAL-2240 and TRP-2239. This study provides a solid theoretical foundation for the development of mTOR inhibitors. Subsequent efforts will focus on optimizing these compounds, targeting structural adjustments to enhance their biological activity and specificity towards mTOR, thereby achieving more precise targeting and treatment of tumors.

## Introduction

The phosphatidylinositol 3-kinase (PI3K)/protein kinase B (Akt) signaling pathway plays a pivotal role in regulating cell growth, metabolism, and survival [1–3]. The mammalian target of rapamycin (mTOR), as the central component of this pathway, exerts a direct influence on cell proliferation and survival, positioning it as a critical therapeutic target in the treatment of cancer and other metabolic diseases [4–7]. Tumorigenesis, driven by abnormal cell proliferation, endows tumor cells with distinctive biological characteristics such as uncontrolled proliferation, sustained angiogenesis, tissue invasion, and metastasis. The overexpression of mTOR is closely linked to the initiation, progression, and malignancy of tumors [8,9].

Sirolimus (rapamycin), the first mTOR inhibitor, was isolated and identified from soil bacteria in the 1970s [10]. Subsequent studies revealed its potent anti-tumor, anti-fungal, and immunosuppressive properties [11–13]. In 1991, the targets of sirolimus, TOR1 and TOR2, were discovered in yeast cells [14]. By 1994, researchers established that mTOR in mammals corresponds to the TOR complexes in yeast cells. In mammals, mTOR exists as part of two distinct complexes: mTOR complex 1 (mTORC1) and mTOR complex 2 (mTORC2). mTORC1 is formed by mTOR, mammalian lethal Sec13 protein 8 (mLST8), PRAS40, and RAPTOR [15], whereas mTORC2 comprises mTOR, mLST8, PRR5, mSIN1, and the rapamycin-insensitive companion of mTOR (RICTOR) [16].

Sirolimus, everolimus, and temsirolimus are targeted therapeutics that primarily inhibit mTORC1, while their effects on mTORC2 are relatively modest [17,18]. As a result, the search for more potent anti-tumor mTOR inhibitors has led researchers to focus on ATP-competitive mTOR inhibitors that can target both mTORC1 and mTORC2 [19,20]. Mao, Beibei et al. synthesized a series of imidazo[1,2-b]pyrazine derivatives by studying the structure-activity relationship of ATP-competitive mTOR inhibitors. Some of these derivatives demonstrated the ability to inhibit the proliferation of A549 cells [21].

Within the field of biopharmaceutical research, identifying targeted drugs for particular proteins has become a key priority, with effective target identification and drug screening technologies being paramount for new drug development [22–24]. By employing a variety of molecular simulation techniques and computational approaches, computer-aided drug design (CADD) greatly enhances the efficiency of screening potential drug molecules. The resolution of three-dimensional structures of many biological macromolecules has rendered structure-based drug design increasingly practical and relevant. By utilizing molecular mechanics and molecular engineering methods, CADD has substantially enhanced the efficiency of drug discovery.

In this study, we report a virtual screening approach, coupled with docking evaluations and molecular dynamics (MD) simulations, aimed at discovering new ATP-competitive mTOR inhibitors. This methodology effectively aids in the identification of hit compounds with promising inhibitory activity against mTOR, thereby validating the reliability of our predictions. Additionally, docking studies have elucidated the ligand-binding conformations of the identified inhibitors, which were further analyzed through MD simulations and binding free energy evaluations, providing a valuable starting point for future lead optimization.

## Virtual screening of the mTOR target

### Compound library preparation

For this docking study, the compound library was predominantly sourced from the ChemDiv commercial compound collection, which consists of 902,998 compounds. Data collection for the compound library preparation was conducted in November 2023. The compounds were processed using the LigPrep module on the Maestro 11.9 platform, undergoing protonation and energy minimization, with the OPLS3e force field applied throughout.

### Preparation of the mTOR target protein structure

The mTOR target protein structure (PDB ID: 4JSX) [19] was meticulously prepared on the Maestro 11.9 platform. This preparation included the removal of water molecules and ions, protonation, addition of missing atoms, completion of missing groups, and subsequent energy minimization and optimization. The OPLS3e force field was utilized for all these steps.

### Molecular docking

The virtual screening and optimization processes were carried out using the Glide module in Schrödinger Maestro software. Protein processing was performed through the Protein Preparation Wizard module, which involved receptor preprocessing, optimization, and minimization, using the OPLS3e force field for constrained minimization. All compounds were prepared with the default settings of the LigPrep module. During the Glide screening process, the prepared receptor was imported to designate the appropriate location for receptor grid generation. Glide generates 32 distinct conformations for each ligand during the preparation phase. The docking site was identified based on the protein’s native ligand, 17G, which served as the centroid for a 15Å x 15Å x 15Å box (coordinates: x=50.24, y=-1.39, z=-47.64). The ligand was initially redocked to validate the chosen docking method.

The HTVS (High-Throughput Virtual Screening) was performed based on a pre-defined scoring function. The top 10% (88,000 compounds) were selected based on their energy scores, narrowing the scope of the large-scale screening and initially identifying potential compounds. The SP docking method refined the initial screening results, selecting the top 10% (8,800 compounds) for further optimization of their binding conformations. The XP docking method precisely screened these 8,800 compounds, and strict scoring was applied to identify tightly binding compounds, ensuring progressively improved screening accuracy and quality. MMGBSA was used to accurately calculate binding energies, and ADME analysis evaluated pharmacokinetic properties. Based on an integrated consideration of energy and drug-likeness potential, 762 compounds were strictly selected. Then, further analysis of hydrogen bond formation, π-π stacking, and hydrophobic interactions was conducted. Finally, 50 compounds were precisely selected based on binding strength, stability, and specificity. The flowchart is shown below.

### Molecular dynamics

In this study, molecular dynamics simulations of the screened receptor protein-ligand complexes were performed using the Gromacs 2020 software package. The AMBER99SB-ILDN force field parameters were applied to the protein, while the generalized AMBER force field (GAFF) parameters were used for the small molecule ligands. The Sobtop program was employed to construct the topology for the small molecules, with RESP charge fitting performed using the B3LYP/6-31G(d) basis set. The TIP3P explicit water model was selected, ensuring a minimum distance of 1.0 nm between any protein atom and the edge of the water box. Based on the docking results, sodium or chloride ions were added to neutralize the system’s charge. The molecular dynamics simulation workflow consisted of four steps: energy minimization, heating, equilibration, and production dynamics simulation. Initially, the protein (and small molecule) heavy atoms were restrained, and the water molecules underwent 10,000 steps of energy minimization (5,000 steps each using the steepest descent and conjugate gradient methods). The restraints were then removed, and the entire system was subjected to another 10,000 steps of energy minimization using the same methods. The system was gradually heated to 300 K over 50 ps. After heating, the system was equilibrated for 50 ps under the NPT ensemble. Finally, a 20 ns molecular dynamics simulation was conducted under the NPT ensemble, with trajectory data saved every 2 ps and analyzed using the trjconv module. The binding free energy between the ligand and protein was calculated using the gmx_MMPBSA method in the Gromacs 2020 program. To conduct an in-depth analysis of molecular dynamics, this study performed RMSD, RMSF, SASA, and Rg analyses.

#### Root mean square deviation (RMSD).

RMSD analysis can indicate whether the simulation has reached equilibrium [25], with convergence being crucial, RMSD values should stabilize around a fixed point [26]. If the protein’s RMSD continues to increase or decrease at the end of the simulation, it suggests the system has not yet equilibrated, and the simulation time may be insufficient for comprehensive analysis. Additionally, RMSD reflects the stability of the protein-small molecule complex; a larger RMSD suggests greater instability in the protein. Select the atomic set for RMSD calculation, define the calculation range, compute the output values, and plot the RMSD vs. time curve.

#### Root mean square fluctuation (RMSF).

RMSF is used to characterize the conformational changes of each amino acid along the protein chain during the simulation, with peaks indicating regions of the greatest fluctuation [27]. A higher RMSF value signifies greater variability in conformational states and increased flexibility of the amino acid residues. Choose the atomic range, set the trajectory range, and run the calculation. After computation, output the RMSF values and analyze the variation in atomic movement amplitude over time or space.

#### Solvent-accessible surface area (SASA).

During protein folding, hydrophobic residues tend to be buried within the molecule. The SASA provides insights into protein folding and hydrophobicity [28]. Set the atomic radius and other parameters, run the calculation, and the software will compute the SASA of each atom based on the molecular conformation and set parameters, then calculate the SASA for the selected region.

#### Radius of gyration (Rg).

Rg, which measures the mass-weighted distance between receptor atoms and the center of mass, serves as an indicator of the protein structure’s compactness. A lower Rg value corresponds to a more compact and stable protein structure, while a higher Rg indicates greater conformational entropy and disorder. Define the atomic range and set the trajectory calculation range; click calculate to obtain the Rg value and plot the variation curve.

#### Free energy landscape (FEL) analysis.

We will construct a FEL based on the principal components analysis (PCA) derived from the trajectory data. This approach will provide insights into the conformational dynamics of the protein-ligand complexes and help identify the most stable conformational states during the MD simulations.

## Results and discussion

### Analysis of binding mode and method validity

**Fig 1:**
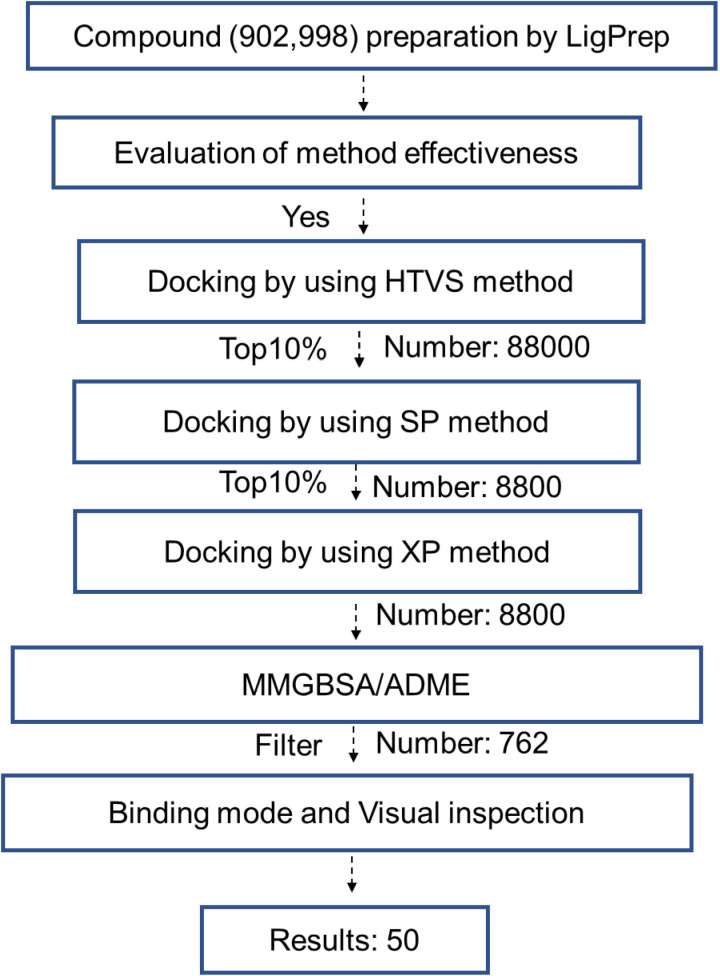
The docking model and analysis of mTOR with target protein. (A) The 3D structure of the mTOR complex is represented with the protein backbone depicted as a tube and highlighted in bright cyan. (B) A detailed view of the active site bound to the ligand. The key residues interacting with the ligand are displayed as sticks and colored in cyan. (C) The 2D interaction diagram of the ligand-mTOR complex, illustrating the protein-ligand interactions. (D) The re-docking results of 17G (red) with mTOR protein.

The crystal structure of the mTOR protein is highly accurate, with no missing key residues, and its active site is well-defined. This active site primarily consists of residues such as VAL-2240, TYR-2225, CYA-2243, MET-2345, TRP-2239, LEU-2185, ILE-2163, PRO-2169, GLU-2190, ILE-2237, and ILE-2356. For this study, 17G, the natural ligand of mTOR, was selected as the reference compound, and its binding mode is illustrated in Fig 1. The 17G ligand forms strong hydrogen bonds with key residues, particularly VAL-2240 (gatekeeper) and GLU-2190 [25]. Additionally, the nitrogen-containing heterocycle and benzene ring of 17G engage in conjugated interactions with residues such as TYR-2225, CYA-2243, MET-2345, TRP-2239, LEU-2185, ILE-2163, PRO-2169, ILE-2237, and ILE-2356. Notably, the benzene ring of 17G establishes pi-pi conjugated interactions with TYR-2225 and TRP-2239, which are crucial for stabilizing the ligand (Fig 1B and C). These interactions underscore the importance of these amino acids during the screening process. To validate the docking protocol for screening potential active compounds, the known native ligand was docked into the mTOR binding site. The resulting binding pose showed excellent overlap with the ligand in the original complex (RMSD < 0.5Å, Fig 1D), confirming that this screening method is both effective and reliable.

### Screening results and visualization analysis

From the docking results, the top 8,800 compounds were selected based on their energy scores. These compounds were further refined using MMGBSA and ADME analyses, focusing on binding energies and interactions with key active site residues, which led to the identification of 50 promising compounds (Table 1).

**Table 1:** Virtual screening of specs compound library for mTOR target.

Name	Score	MMGBSA	HA	HD	RN	logP	MW
**F882-0316**	−10.14	−73.72	4	2	8	3.27	446.53
**F471-0162**	−10.14	−72.57	4	1	8	4.71	433.51
**F524-0181**	−10.05	−72.51	6	2	6	3.65	449.49
**L115-0329**	−10.08	−72.49	5	0	7	3.85	408.48
**E898-2215**	−10.89	−72.18	4	1	8	3.65	458.85
**F471-0740**	−10.13	−71.95	4	1	6	4.01	423.45
**J021-1349**	−10.49	−70.10	7	3	9	1.69	413.49
**F471-0751**	−10.18	−69.96	4	1	6	3.86	405.46
**L460-0007**	−10.53	−69.84	3	0	6	4.42	388.51
**L429-0755**	−10.15	−69.60	5	1	10	3.18	460.49
**E898-2261**	−10.86	−69.55	4	1	6	3.76	444.82
**E898-2280**	−10.91	−68.59	4	1	6	3.47	424.41
**L460-0006**	−10.74	−68.43	3	0	6	3.55	360.46
**E898-2229**	−10.63	−68.17	4	1	7	3.61	424.41
**E898-2340**	−10.27	−67.99	4	1	6	3.62	448.79
**C200-7282**	−10.19	−67.94	4	2	5	3.30	369.42
**M455-0443**	−10.39	−67.77	6	0	6	3.96	416.44
**E898-2234**	−11.01	−67.60	4	1	6	2.99	414.34
**E898-2438**	−10.95	−67.58	4	1	8	1.77	414.37
**E898-2309**	−11.41	−67.52	4	1	7	3.12	428.37
**F980-0796**	−10.67	−67.34	5	0	7	0.86	459.93
**F471-0747**	−10.24	−67.28	4	1	6	4.19	419.48
**F471-0747**	−10.24	−67.28	4	1	6	4.19	419.48
**C200-7170**	−10.10	−67.00	4	2	6	3.18	357.41
**5137-3646**	−10.15	−66.98	3	0	1	4.27	354.86
**E898-2413**	−11.13	−66.92	4	1	7	3.12	428.37
**M177-0685**	−10.01	−66.86	4	0	4	3.60	387.37
**F882-0418**	−10.14	−66.58	5	2	7	2.94	446.49
**L977-1593**	−10.80	−66.40	3	1	4	3.23	418.52
**Y020-0211**	−10.44	−66.35	4	0	3	2.65	368.24
**4072-2785**	−10.04	−66.31	5	0	3	3.38	380.47
**8629-0178**	−10.29	−66.27	5	1	5	2.84	395.44
**L977-1399**	−10.26	−65.74	3	1	3	3.05	424.91
**8018-2017**	−10.16	−65.72	5	0	4	1.85	370.45
**L977-0711**	−10.21	−65.66	3	1	4	4.30	400.48
**L977-1429**	−10.89	−65.46	4	2	8	3.19	404.47
**T508-0034**	−10.52	−65.28	4	1	6	1.10	367.45
**L414-0588**	−10.37	−65.07	3	1	Table	3.17	369.45
**L977-1242**	−10.71	−65.03	4	2	7	3.01	410.86
**G818-0020**	−10.46	−64.89	4	3	3	2.61	303.32
**F472-0015**	−10.04	−64.84	5	2	11	4.14	492.60
**F389-0036**	−10.13	−64.74	2	2	5	3.65	394.81
**4072-2778**	−10.01	−64.63	3	0	1	3.68	320.42
**F410-1237**	−10.19	−64.57	2	2	4	4.67	351.41
**3235-0086**	−10.12	−64.53	4	1	1	2.93	322.39
**7623-0087**	−10.21	−64.47	3	1	3	3.14	335.39
**M115-0203**	−10.43	−64.43	6	0	5	3.42	404.43
**L977-1200**	−10.04	−64.42	3	1	3	3.86	400.48
**L977-1456**	−11.06	−64.41	4	3	6	2.60	390.44
**8018-0713**	−10.64	−63.98	4	1	4	3.83	346.77

Name, the compound’s exclusive identifier in the ChemDiv library; Score, binding ability; MMGBSA, binding energy value; HA, number of hydrogen bonds; HD, number of hydrogen bond donors; RN, screening rank; logP, lipophilicity/hydrophilicity balance; MW, stands for molecular weight.

**Fig 2:**
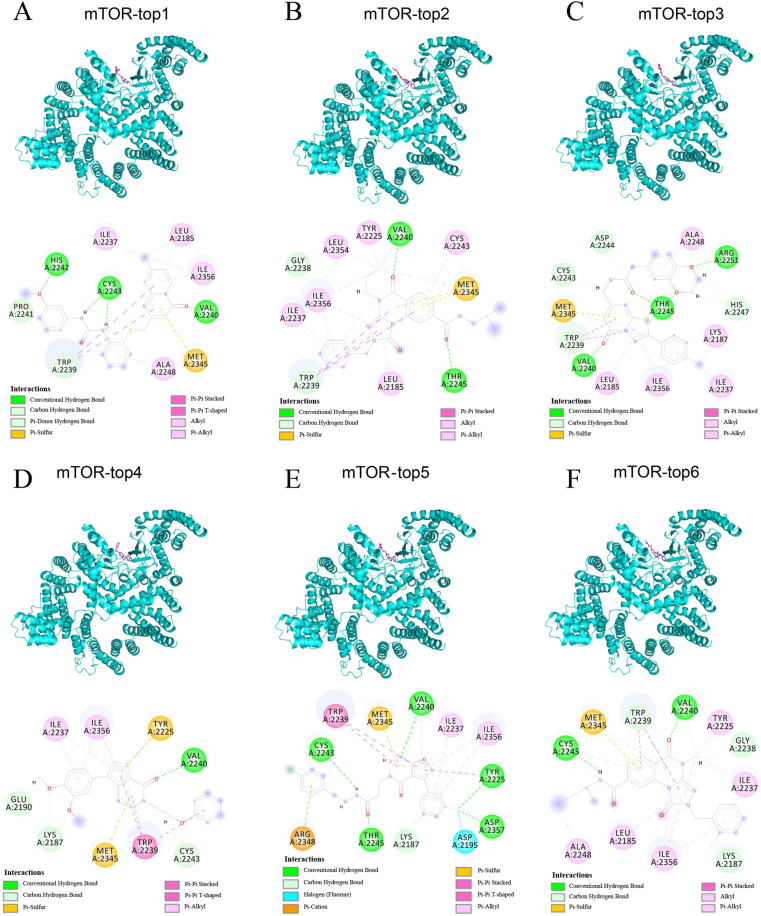
The detail binding mode of mTOR with top1, top2, top3, top4, top5, top6.

Among these, six compounds (top1, top2, top3, top4, top5, top6) were chosen for detailed visualization studies (Fig 2). These selected compounds demonstrated strong binding affinity with the mTOR target protein, all exhibiting binding energies below -10 kcal/mol, indicating a high level of specificity and compatibility. The compound-protein complexes were visualized using PyMOL 2.1 software to explore the binding modes in detail. The visualization clearly outlines the interactions between the compounds and the protein’s binding pocket, including the specific amino acid residues involved. For example, top1 formed multiple hydrogen bonds with the mTOR protein’s active site residues (VAL-2240, CYS-2243, HIS-2242). Moreover, the compound’s nitrogen-containing heterocycle and benzene ring engage in conjugated interactions with residues like ILE-2237, LEU-2185, ILE-2356, ALA-2248, TRP-2239, and MET-2345. Notably, a strong pi-pi conjugated interaction with TRP-2239 was observed, which enhanced the molecule’s stability at the protein’s active site, potentially increasing its biological activity (Fig 2A).

The top2 compound formed robust hydrogen bonds with the VAL-2240 and THR-2245 residues at the protein’s binding site. Additionally, its benzene ring engaged in a strong pi-pi interaction with the TRP-2239 residue, significantly contributing to the stability of the small molecule within the protein’s cavity (Fig 2B). Top3 displayed hydrogen bonding with critical residues, including VAL-2240, THR-2245, and ARG-2251, along with hydrophobic interactions involving ALA-2248, LYS-2187, ILE-2237, ILE-2356, LEU-2185, MET-2345, and TRP-2239. These interactions collectively enhanced the stability of the small molecule-protein complex. Furthermore, the nitrogen-containing ring of the compound formed a pi-pi interaction with the TRP-2239 benzene ring, further stabilizing the complex (Fig 2C). Top4 showed a strong affinity for the mTOR target, forming a critical hydrogen bond with the VAL-2240 residue, which is essential for anchoring the small molecule within the protein pocket. This interaction, coupled with the compound’s hydrophobic interactions with surrounding residues, suggests a stable and effective binding (Fig 2D). Top5 formed multiple hydrogen bonds with key residues such as VAL-2240, TYR-2225, ASP-2357, THR-2345, and CYS-2243. The compound also participated in pi-pi and pi-cation interactions with TRP-2239 and ARG-2348, respectively, which are vital for the formation of a stable complex (Fig 2E). Top6 was characterized by hydrogen bonding with VAL-2240 and CYS-2243, as well as a pi-pi interaction with TRP-2239, all of which are crucial for the stability of the small molecule-protein complex (Fig 2F).

Comparative analysis of these six compounds reveals that each effectively formed hydrogen bonds with the gatekeeper residue VAL-2240 and engaged in pi-pi interactions with TRP-2239. Additionally, their hydrophobic interactions with the protein’s pocket residues closely mimic the binding mode of a known active compound, indicating their potential as potent mTOR inhibitors.

### Molecular dynamics validation analysis

To further investigate the interactions between small molecules and the protein, we conducted a 20 ns molecular dynamics simulation of the mTOR protein in complex with the small molecules top1, top2, top3, top4, top5, and top6. Monitoring the protein’s RMSD throughout the simulation offers deeper insights into its structural conformations [26].

**Fig 3:**
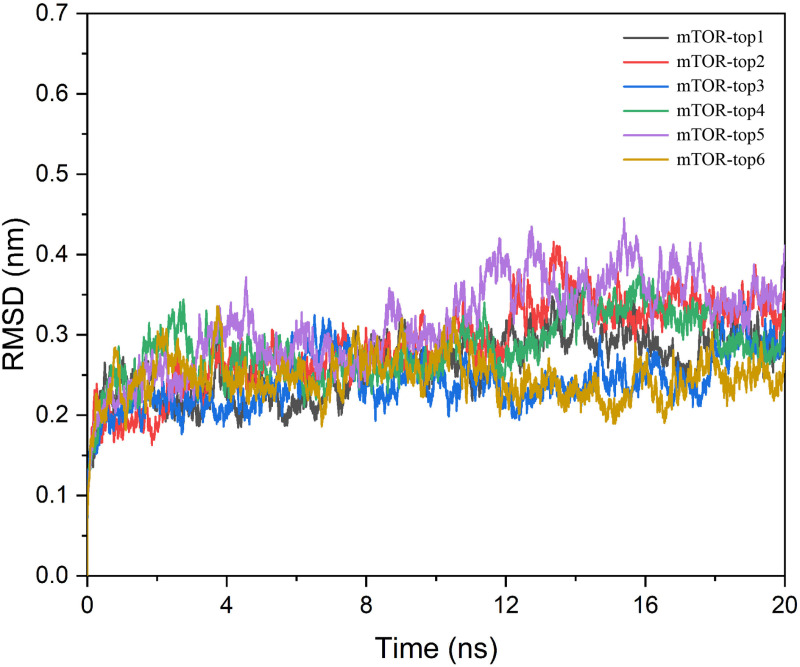
The RMSD of six compounds with mTOR protein.

As shown in Fig 3, the average RMSD of the mTOR protein in complex with the six compounds was less than 3.5 Å, and the complexes achieved dynamic equilibrium within 2 ns. This indicates that the small molecules are well-suited to the protein target, forming stable complexes. Moreover, no significant structural discontinuities were observed in the conformational changes of the complexes, further confirming that the small molecules bind effectively with the protein and remain within the active pocket. However, when comparing the RMSD fluctuations among the six complexes, we observed that mTOR-top2, mTOR-top4, and mTOR-top5 exhibited slightly larger fluctuations. This may be due to the presence of unfavorable contacts in the binding modes during docking, which were subsequently resolved as new equilibria were established following dynamics adjustments.

**Fig 4:**
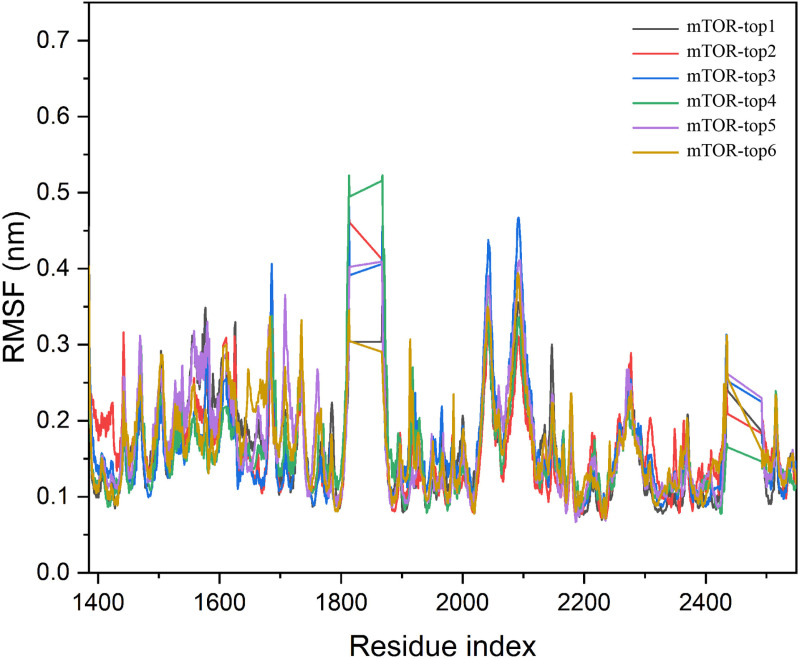
The RMSF of six compounds with mTOR protein.

**Fig 5:**
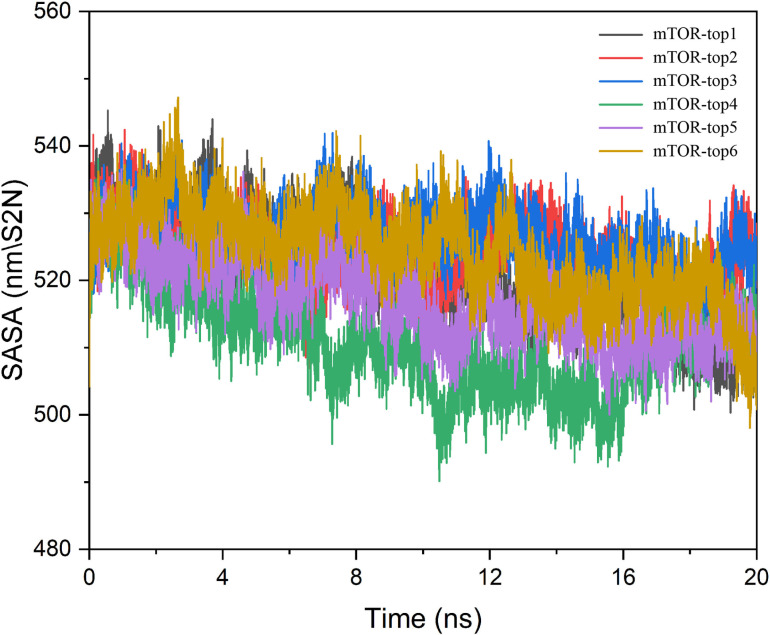
The SASA of six compounds with mTOR protein.

As illustrated in Fig 4, only a few amino acid residues showed significant conformational changes within the protein-small molecule complexes. This is primarily because these residues are located in the protein’s hinge regions, which are more flexible and susceptible to conformational changes during the simulation. Most amino acid residues exhibit minimal fluctuation, reflecting the stability of the small molecule-protein complexes. Furthermore, the binding sites of the small molecules are concentrated around residues 2200–2300, which display minimal fluctuation. This likely contributes to the stability of the interactions between the small molecules and the protein. Surface characteristics of proteins are crucial for understanding protein structure and biological function. SASA measures the surface area of a biomolecule accessible to the solvent. As shown in Fig 5, there is a notable decrease in the accessible surface area of the complexes, indicating that the binding of the small molecules does not compromise the stability of the protein itself. This further supports the conclusion that the compounds bind effectively to the protein.

**Fig 6:**
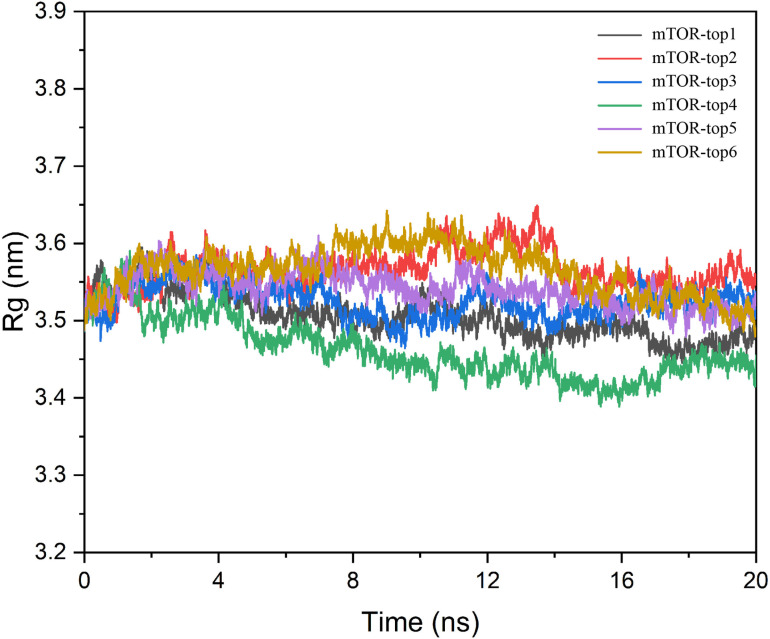
The Rg of six compounds with mTOR protein.

In this study, we aimed to analyze the compactness and stability of protein-compound complexes induced by binding at primary and secondary sites, using the Rg as a key metric [29]. From the Rg analysis, we observed a general reduction across all proteins, suggesting that the interaction with the compounds during molecular dynamics simulations led to enhanced electrostatic and hydrophobic contacts, thereby stabilizing the complexes (Fig 6). Among these, the mTOR-top1 complex exhibited a marked decrease in Rg, indicative of significant protein contraction. This phenomenon may be attributed to an unfavorable binding mode of the small molecule within the protein cavity. Further examination of the dynamic conformations revealed a slight dissociation of the small molecule from the protein pocket, which could negatively impact the compound’s activity, highlighting the need to avoid such scenarios in future experiments.

**Fig 7:**
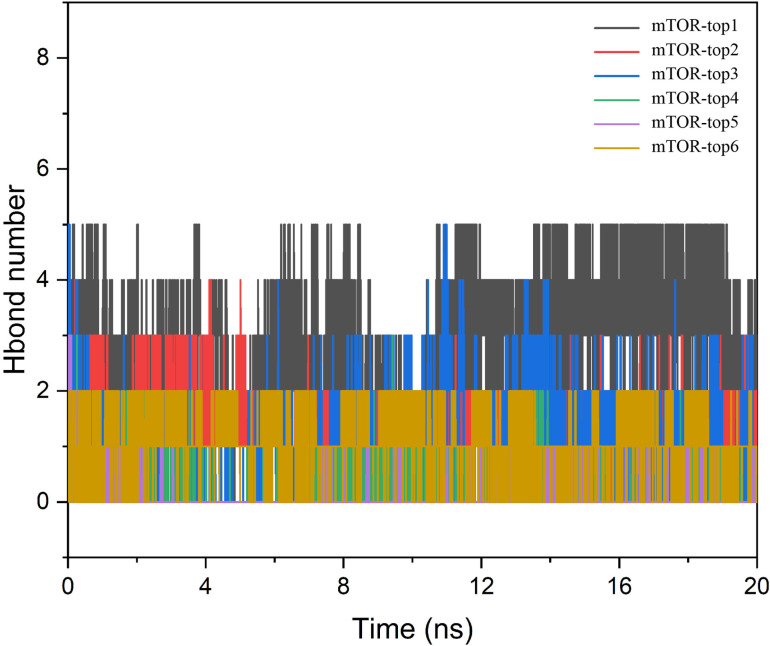
The hydrogen bond number of six compounds with mTOR protein.

Furthermore, during the simulations, the ligand formed several hydrogen bonds with the protein, underscoring the effectiveness of molecular dynamics in characterizing hydrogen bond interactions [28]. To gain a clearer understanding of the binding interactions between the protein and the compounds, we analyzed the variations in hydrogen bond numbers throughout the simulation. The hydrogen bond network diagrams for each protein-compound pair revealed that mTOR-top1, mTOR-top2, mTOR-top3, and mTOR-top6 formed stable hydrogen bonds with amino acids within the protein pocket. These interactions are crucial for stabilizing the protein-ligand complex. Conversely, other compounds displayed a loss of hydrogen bonds due to their dissociation from the protein’s core active site region during the dynamics (Fig 7).

**Fig 8:**
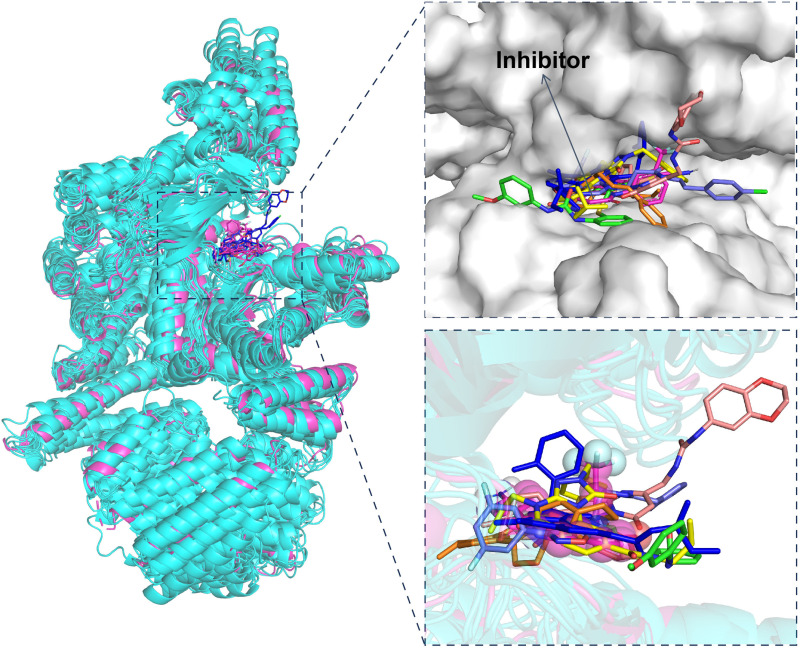
Superposition of six compounds with the mTOR protein (cyan) and the crystal mTOR structure (magenta). The compounds are rendered in green (top1), yellow (top2), light pink (top3), orange (top4), slate (top5), blue (top6), and magenta (ATP-site inhibitor), respectively.

**Fig 9:**
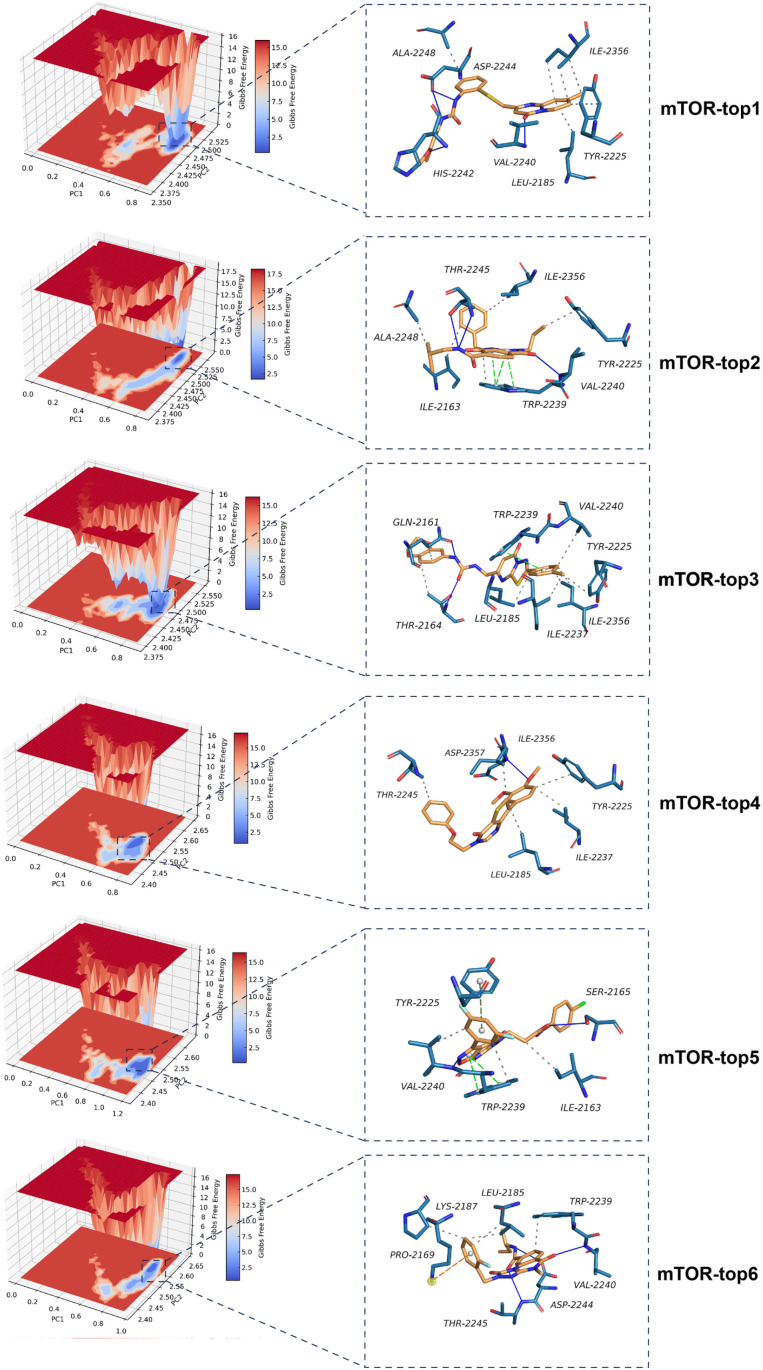
The binding modes of six compounds with the mTOR protein.

To ensure that the compounds are indeed interacting with the correct active site, we performed a structural overlay of the six most stable conformations of the compounds with a protein structure that contains a known natural ligand. This reference structure was selected because it represents the natural binding pocket for the ligand in the mTOR protein. From the structural alignment results, we observe that all six compounds effectively bind to the same active site as the ATP-site inhibitor (Fig 8). This binding site is consistent with the known functional region of the mTOR protein, supporting the conclusion that the compounds are potential inhibitors by occupying the correct active site. Moreover, we employed conformational dynamics to gain a deeper understanding of the interactions between the compound and the active site (Fig 9). And ADMET predictions were performed, and the detailed results can be found in the supplementary materials. We believe these additional validation enhances the reliability of our findings and better demonstrates the potential of these compounds as effective inhibitors.

Binding free energy, calculated as ΔGbinding energy, is a critical tool for analyzing the thermodynamic stability of ligand binding [30]. A negative binding free energy value signifies system stability, whereas a positive value indicates instability (Table 2). The data from Table 2 reveal that van der Waals forces are the primary contributors to the stabilization of the small molecules, followed closely by electrostatic interactions. Notably, the compounds mTOR-top1, mTOR-top2, and mTOR-top6 demonstrated the most favorable binding interactions with the mTOR protein. For example, the binding free energy of top1 with mTOR was -123.103±11.869 kJ/mol, with van der Waals forces being the dominant stabilizing factor (-199.811±7.553 kJ/mol). This suggests that the compound is effectively stabilized within the protein pocket through robust van der Waals interactions with surrounding residues. This is largely due to the compound’s deep penetration into the protein cavity, which enables strong van der Waals interactions with adjacent amino acids. Additionally, the formation of hydrogen bonds between the compound and the protein pocket contributes to the small molecule’s stability through significant electrostatic interactions (−69.801±9.596 kJ/mol).

**Table 2:** The binding energy by MMGBSA (kJ/mol).

Type	mTOR-top1	mTOR-top2	mTOR-top3	mTOR-top4	mTOR-top5	mTOR-top6
***E***_***VDW***_	−199.811 ± 7.553	−213.853 ± 9.786	−172.016 ± 7.78	−207.458 ± 11.473	−202.553 ± 10.63	−196.376 ± 13.954
***E***_***ELE***_	−69.801 ± 9.596	−18.156 ± 4.283	−28.681 ± 10.594	−7.697 ± 6.095	5.777+/−8.646	−20.221 ± 6.839
***E***_***GB***_	167.188 ± 11.359	104.676 ± 9.819	96.14 ± 9.867	134.12 ± 13.985	104.909 ± 13.064	116.509 ± 11.689
***E***_***SA***_	−20.679 ± 0.841	−20.891 ± −1.104	−18.873 ± −1.01	−22.323 ± 1.294	−20.08 ± 0.802	−19.467 ± 1.154
***G***_***binding energy***_	−123.103 ± 11.869	−148.224 ± 11.986	−123.43 ± 10.888	−103.358 ± 15.243	−111.947 ± 11.475	−119.554 ± 13.615

*E*_*VDW*_, van der Waals energy; *E*_*ELE*_, eletrostatic energy; *E*_*GB*_, polar contribution to solvation; *E*_*SA*_, non-polar contribution to solvation.

Conversely, in the mTOR-top3, mTOR-top4, and mTOR-top5 complexes, the small molecules tended to dissociate from the core active site of the protein, particularly in the mTOR-top4 and mTOR-top5 complexes, where hydrogen bond interactions with the protein were nearly nonexistent. This significantly reduced the efficacy of these small molecules. Although the mTOR-top3 complex displayed some hydrogen bond interactions, it failed to establish hydrogen bonds with the crucial residue VAL-2240, which hinders the compound’s ability to effectively exert its biological activity.

Overall, in the S1 Table, we have summarized the parameters for different tests to optimize and simplify the selection of compounds.

## Conclusion

This study employed virtual screening combined with docking evaluation and molecular dynamics simulations to investigate ATP-competitive mTOR inhibitors. From the ChemDiv commercial compound library, 50 compounds with strong binding modes to the mTOR protein and favorable docking scores were selected. Among them, top1, top2, and top6 exhibited outstanding stability in the active region of the protein during molecular dynamics simulations. These compounds formed strong hydrogen bonds and π-π conjugation interactions with key residues VAL-2240 and TRP-2239, and demonstrated significant hydrophobic interactions. These findings provide a theoretical foundation for the development of mTOR inhibitors. Based on this, further optimization of these compounds will be carried out to enhance their regulatory specificity for mTOR biological activity, facilitating the transition of “old drugs” to a new generation of “new drugs.”

## Supporting information

S1 Table Comparison of six compounds under different tests/parameters. (DOCX)

S1 File ADMET. (CSV)
