# Supplementary material for: Virtual screening and molecular dynamics simulation study of ATP-competitive inhibitors targeting mTOR protein
Source: PLoS One. 2025 May 5;20(5):e0319608. doi: 10.1371/journal.pone.0319608 (PMC12052163; doi:10.1371/journal.pone.0319608)
Supplement: S1 Table — (DOCX) [file pone.0319608.s001.docx]

**S1 Table. Comparison of six compounds under different tests/parameters.**

| **Compound Name** | **Binding Energy (kcal/mol)** | **Hydrogen Bond Formation** | **Average RMSD (Å)** | **Number of Residues with Significant RMSF Variation** | **SASA Change Trend** | | **Average Rg** | **Binding Free Energy (kJ/mol)** | |
| --- | --- | --- | --- | --- | --- | --- | --- | --- | --- |
| **Top1** | -10.14 | Forms hydrogen bonds with VAL-2240, CYS-2243, HIS-2242, etc.; π-π conjugation with TRP-2239 | Less than 3.5 (dynamic equilibrium reached within 2 ns) | Few | Significantly decreased | Decreased | | -123.103±11.869 (ΔGbinding energy) |  |
| **Top2** | -10.14 | Forms hydrogen bonds with VAL-2240, THR-2245; π-π interaction with TRP-2239 | Less than 3.5 (dynamic equilibrium reached within 2 ns) | Few | Significantly decreased | No significant change observed | | -148.224±11.986 (ΔGbinding energy) |  |
| **Top3** | -10.05 | Forms hydrogen bonds with VAL-2240, THR-2245, ARG-2251, etc.; π-π interaction with TRP-2239 | Less than 3.5 (dynamic equilibrium reached within 2 ns) | Few | Significantly decreased | No significant change observed | | -123.43±10.888 (ΔGbinding energy) |  |
| **Top4** | -10.53 | Forms key hydrogen bonds with VAL-2240 | Less than 3.5 (dynamic equilibrium reached within 2 ns) | Few | Significantly decreased | No significant change observed | | -103.358±15.243 (ΔGbinding energy) |  |
| **Top5** | -10.89 | Forms hydrogen bonds with VAL-2240, TYR-2225, ASP-2357, THR-2345, CYS-2243, etc.; π-π interaction with TRP-2239 | Less than 3.5 (dynamic equilibrium reached within 2 ns) | Few | Significantly decreased | No significant change observed | | -111.947±11.475 (ΔGbinding energy) |  |
| **Top6** | -10.91 | Forms hydrogen bonds with VAL-2240, CYS-2243; π-π interaction with TRP-2239 | Less than 3.5 (dynamic equilibrium reached within 2 ns) | Few | Significantly decreased | No significant change observed | | -119.554±13.615 (ΔGbinding energy) |  |
